# The European Society of Gynaecological Oncology (ESGO), the International Society for the Study of Vulvovaginal Disease (ISSVD), the European College for the Study of Vulval Disease (ECSVD) and the European Federation for Colposcopy (EFC) Consensus Statements on Pre-invasive Vulvar Lesions

**DOI:** 10.1097/LGT.0000000000000683

**Published:** 2022-06-21

**Authors:** Mario Preti, Elmar Joura, Pedro Vieira-Baptista, Marc Van Beurden, Federica Bevilacqua, Maaike C. G. Bleeker, Jacob Bornstein, Xavier Carcopino, Cyrus Chargari, Margaret E. Cruickshank, Bilal Emre Erzeneoglu, Niccolò Gallio, Debra Heller, Vesna Kesic, Olaf Reich, Colleen K. Stockdale, Bilal Esat Temiz, Linn Woelber, François Planchamp, Jana Zodzika, Denis Querleu, Murat Gultekin

**Affiliations:** 1 Department of Surgical Sciences, University of Torino, Torino, Italy; 2 Department of Gynecology and Gynecologic Oncology, Comprehensive Cancer; Center, Medical University of Vienna, Vienna, Austria; 3 Hospital Lusiadas Porto, Porto, Portugal; Lower Genital Tract Unit, Centro Hospitalar de São João, Porto, Portugal; 4 Centre for Gynecological Oncology Amsterdam, Netherlands Cancer Institute/Antoni van Leeuwenhoek Hospital, Amsterdam, The Netherlands; 5 Department of Pathology, Amsterdam UMC, Vrije Universiteit Amsterdam, Cancer Center Amsterdam, Amsterdam, The Netherlands; 6 Galilee Medical Center and Azrieli Faculty of Medicine, Bar-Ilan, Israel; 7 Department of Obstetrics and Gynaecology, Hôpital Nord, APHM, Aix-Marseille University (AMU), Univ Avignon, CNRS, IRD, IMBE UMR 7263, 13397, Marseille, France; 8 Radiation Therapy, Gustave Roussy Cancer Campus, Paris, France; 9 Aberdeen Centre for Women’s Health Research, University of Aberdeen, Aberdeen, United Kingdom; 10 Faculty of Medicine, Department of Obstetrics and Gynecology, Division of Gynaecological Oncology, Hacettepe University, Ankara, Turkey; 11 Rutgers New Jersey Medical School, Newark, NJ; 12 Department of Obstetrics and Gynecology, University of Belgrade, Belgrade, Serbia; 13 Department of Obstetrics and Gynecology, Medical University of Graz, Graz, Austria; 14 Department of Obstetrics & Gynecology, University of Iowa, Iowa City, IA; 15 Department of Gynecology, Hamburg-Eppendorf University Medical Center, Dysplasia Center Hamburg, Jerusalem Hospital, Hamburg, Germany; 16 Clinical Research Unit, Institut Bergonie, Bordeaux, France; 17 Department of Obstetrics and Gynaecology Rīga Stradiņš university, Riga, Latvia; 18 Department of Obstetrics and Gynecologic Oncology, University Hospital, Strasbourg, France; Division of Gynecologic Oncology, Fondazione Policlinico Universitario A Gemelli IRCCS, Rome, Italy; 19 Division of Gynaecological Oncology, Department of Obstetrics and Gynaecology, Hacettepe University Faculty of Medicine, Ankara, Turkey

## Abstract

The European Society of Gynaecological Oncology (ESGO), the International Society for the Study of Vulvovaginal Disease (ISSVD), the European College for the Study of Vulval Disease (ECSVD), and the European Federation for Colposcopy (EFC) developed consensus statements on pre-invasive vulvar lesions in order to improve the quality of care for patients with vulvar squamous intraepithelial neoplasia, vulvar Paget disease in situ, and melanoma in situ. For differentiated vulvar intraepithelial neoplasia (dVIN), an excisional procedure must always be adopted. For vulvar high-grade squamous intraepithelial lesion (VHSIL), both excisional procedures and ablative ones can be used. The latter can be considered for anatomy and function preservation and must be preceded by several representative biopsies to exclude malignancy. Medical treatment (imiquimod or cidofovir) can be considered for VHSIL. Recent studies favor an approach of using imiquimod in vulvar Paget’s disease. Surgery must take into consideration that the extension of the disease is usually wider than what is evident in the skin. A 2 cm margin is usually considered necessary. A wide local excision with 1 cm free surgical margins is recommended for melanoma in situ. Following treatment of pre-invasive vulvar lesions, women should be seen on a regular basis for careful clinical assessment, including biopsy of any suspicious area. Follow-up should be modulated according to the risk of recurrence (type of lesion, patient age and immunological conditions, other associated lower genital tract lesions).

## BACKGROUND

The European Society of Gynaecological Oncology (ESGO), the International Society for the Study of Vulvovaginal Disease (ISSVD), the European College for the Study of Vulval Disease (ECSVD), and the European Federation for Colposcopy (EFC) are leading international societies among gynecologists, pathologists, dermatologists, and related disciplines. One of their aims is to promote the highest quality of care for women with pre-invasive and invasive gynecological neoplasia through prevention, advancing treatment, excellence in care, and high-quality research and education.

ECSVD, EFC, ESGO, and ISSVD collaborated to develop a consensus statement on pre-invasive vulvar lesions.

## METHODS

The ESGO, ISSVD, ECSVD, and EFC executive councils nominated selected specialists from their membership bodies with well-recognized expertise, clinical and research activity, and leadership in the field as surrogate markers for their continuous effort in improving the quality of care for patients with vulvar and vaginal pre-invasive lesions.

A systematic literature review of studies published from January 2000 to March 2021 was carried out using the MEDLINE database. Search indexing terms and criteria are listed in an additional file (see Supplemental Digital Content 1, http://dx.doi.org/10.1136/ijgc-2021-003262). The literature search was limited to publications in English, Italian, Spanish, Portuguese, German, and French. The search strategy excluded editorials, case reports, letters, and in vitro studies.

A total number of 192 articles were retrieved; 89 were on squamous vulvar intraepithelial neoplasia (VIN), 33 on vulvar Paget’s disease, and 26 on vulvar melanoma in situ. A further 12 articles with more than one pre-invasive disease and 32 reviews were considered.

Data extraction was performed for all articles dealing with treatment by two independent teams and was double-checked. Tables with the most relevant clinical outcomes were completed and summarized in the text (see Supplemental Digital Content 2 and 3, http://dx.doi.org/10.1136/ijgc-2021-003262).

Evidence-based consensus statements were also developed on the management of patients with pre-invasive vulvar lesions, chaired by professors Mario Preti and Murat Gultekin. The chairs were responsible for drafting corresponding preliminary statements based on the review of the relevant literature (residents assisted in preparing data extraction and analyses: F.B., N.G., B.E.E., B.E.T.). These were then sent to the group of selected specialists. A first round of binary voting (agree/disagree) was carried out for each potential statement. The participants took part in each vote, but they were permitted to abstain from voting if they felt they had insufficient expertise to agree/disagree with the statement or if they had a conflict of interest that could be considered to influence their vote. The voters had the opportunity to provide comments/suggestions with their votes. The chairs then discussed the results of this first round of voting and revised the statements if necessary. The voting results and the revised version of the statements were again sent to the whole group, and another round of binary voting was organized according to the same rules, to allow the whole group to evaluate the revised statements. The statements were finalized based on the results of this second round of voting. The group achieved consensus on 12 statements. One of the authors (F.P.) provided the methodology support for the entire process and did not participate in voting for statements.

Two external independent reviewers (M.V.B., M.B.), who have been internationally acknowledged for their research in vulvar preinvasive lesions, reviewed the final manuscript.

### Evolution of Terminology and Classification

The two carcinogenic pathways of vulvar squamous cell neoplasia were reflected in the 1986^1^ and again in the 2004^2^ ISSVD classifications. They included two vulvar intraepithelial neoplasia (VIN) groups: ‘VIN, usual type, HPV related’ and ‘VIN, differentiated type, HPV unrelated’.

The 2013 Lower Anogenital Squamous Terminology (LAST) unifies the nomenclature of human papillomavirus (HPV)-associated squamous lesions of the entire lower anogenital tract and uses a two-tier terminology: ‘low-grade squamous intraepithelial lesion (LSIL)’ and ‘high-grade squamous intraepithelial lesion (HSIL)’ for the vulva as well as other genital organs.^3^ The absence of reference to differentiated vulvar intraepithelial neoplasia (dVIN), despite its malignant potential, and the inclusion of vulvar LSIL (low-grade squamous intraepithelial lesion), recreating the potential for overdiagnosis and overtreatment of benign and usually self-limiting lesions, are the main limitations of the LAST classification.

The 2018 *International classification of diseases for mortality and morbidity statistics*, 11th revision (ICD-11) system^4^ still uses the term ‘carcinoma in situ’ of the vulva for both squamous and non-squamous pre-invasive lesions (Paget’s disease), where the implication of impending cancer may lead to unnecessary radical excisions of every intraepithelial neoplastic lesion.

**Box 1.** 2015 International Society for the Study of Vulvovaginal Disease Terminology of Vulvar Squamous Intraepithelial Lesions

LSIL of the vulva (vulvar LSIL, flat condyloma, or HPV effect)

HSIL of the vulva (vulvar HSIL ((VHSIL)), VIN usual type)

dVIN

dVIN, differentiated-type vulvar intraepithelial neoplasia; HPV, human papillomavirus; HSIL, high-grade squamous intraepithelial lesion; LSIL, lowgrade squamous intraepithelial lesion; SIL, squamous intraepithelial lesion; VIN, vulvar intraepithelial neoplasia.

The current 2015 ISSVD terminology does contain the terms LSIL (low-grade squamous intraepithelial lesion) and HSIL (high-grade squamous intraepithelial lesion) (box 1)^5^; however, the word ‘neoplasia’ was replaced by ‘lesion’, and it was stated that the meaning of LSIL (low-grade squamous intraepithelial lesion) was the manifestation of a productive HPV infection, a flat condyloma, or HPV effect. ‘Vulvar intraepithelial neoplasia differentiated’ was the third category, just as in the previous ISSVD terminologies.

The World Health Organization (WHO) in 2014 used LSIL (lowgrade squamous intraepithelial lesion), HSIL (high-grade squamous intraepithelial lesion), and ‘VIN-differentiated type’,^6^ while the 2020 WHO classification of tumors^7^ divides the vulvar lesions into ‘HPV-associated squamous intraepithelial lesions’ and ‘HPV- independent VIN’ (box 2). Along with dVIN, differentiated exophytic vulvar intraepithelial lesion (DEVIL) and vulvar acanthosis with altered differentiation (VAAD) have been described as subtypes of HPV-independent VIN.

In 1986, the ISSVD classified vulvar Paget’s disease as an *in situ* adenocarcinoma of the vulvar skin.^1^ In 2001, Wilkinson et al proposed a histopathological classification of vulvar Paget’s disease that distinguished primary, of cutaneous origin, vulvar Paget’s disease (type 1) as arising within the vulvar epithelium, from secondary/non-cutaneous vulvar Paget’s disease (type 2), that originates from the spread of an internal malignancy (anorectal adenocarcinoma or urothelial carcinoma of the bladder or urethra, to the vulvar epithelium).^8^ Type 1 vulvar Paget’s disease is further divided into 1a-intraepithelial, 1b-invasive, and 1c-manifestation of an underlying vulvar adenocarcinoma. Vulvar Paget’s disease is a subset of extramammary Paget’s disease.

**Box 2.** 2020 WHO Terminology

**HPV-associated squamous intraepithelial lesions:** low-grade squamous intraepithelial lesion of the vulva (LSIL); high-grade squamous intraepithelial lesion of the vulva (HSIL)

**HPV-independent VIN:** differentiated vulvar intraepithelial neoplasia (dVIN); differentiated exophytic vulvar intraepithelial lesion (DEVIL); vulvar acanthosis with altered differentiation (VAAD)

Even if the 2014 WHO tumors classification^6^ no longer supports Wilkinson classification, current literature often refers to that classification, mainly based on the histopathologic features of vulvar Paget’s disease. The 2014 WHO tumors classification defines vulvar Paget’s disease as intraepithelial neoplasm of epithelial origin expressing apocrine or eccrine glandular-like features and characterized by distinctive large cells with prominent cytoplasm, referred to as Paget cells. This definition was reiterated by the 2020 WHO tumors classification that considers vulvar Paget’s disease an in situ adenocarcinoma of the vulvar skin, with or without underlying invasive adenocarcinoma.^7^ Secondary involvement of vulvar skin by carcinoma of rectal, bladder, and cervical origin is defined as ‘secondary Paget disease’.

Melanoma in situ was originally included in the 1986 ISSVD classification as non-squamous intraepithelial neoplasia.^1^ Cutaneous melanoma is staged using the American Joint Committee on Cancer melanoma staging system for melanoma of the skin.^9^

This staging system has been validated for vulvar melanoma and melanoma in situ. Melanoma in situ represents stage Ia.

### Epidemiology

Vulvar condyloma/condylomatous low-grade squamous intraepithelial lesions (LSIL) are usually associated with low-risk HPV infections (HPV 6 or 11 in 90% of cases).^10^ They do not progress to invasive cancer and are common in the general population with a prevalence of around 107–229 per 100 000 women.^11,12^

Vulvar high-grade squamous intraepithelial lesions (VHSIL) are seen with an incidence of 2.5 to 8.8 per 100 000 women/year and may have a risk of transforming into an invasive carcinoma.^10,13,14^

Differentiated vulvar intraepithelial neoplasia (dVIN) represent less than 10% of all the squamous vulvar intraepithelial lesions^15,16^ and has potential for malignant transformation greater than that of VHSIL (32.8% in elderly women with dVIN vs 5.7% in VHSIL seen in young patients).^17^ In a recent Dutch study, the overall European Standardized Rate of high-grade VIN without concurrent vulvar squamous cell carcinoma was 2.99 per 100 000 woman-years: 2.95 for VHSIL and 0.05 for dVIN. This rate has increased for VHSIL from 2.39 between 1991–1995 to 3.26 between 2006–2011 (+36.4%) and from 0.02 to 0.08 (+300.0%) for dVIN.^15^ Using the Surveillance, Epidemiology, and End Results (SEER) databases, between 1973 and 2004, the incidence of VIN and vulvar squamous cell carcinoma increased 3.5% and 1.0% per year, respectively, in the USA, and the largest increase was seen in younger patients.^18^

Despite the rarity of anal cancer at the population level (1–2 cases per 100 000 person-years), due to the HPV field infection, VHSIL patients are at increased risk for anal squamous cell carcinoma and precursors. A recent meta-analysis showed an incidence ratio of anal cancer of 42 per 100 000 person-years (95% CI 33 to 52) in women diagnosed with VHSIL,^19^ that is the third-highest risk for anal cancer after HIV-positive men who have sex with men ≥30 years old and transplanted women ≥10 years post-transplant. The mean time interval between the incidence of VIN and anal cancer diagnosis was 8.9 years.^20^

Extramammary Paget’s disease accounts for about 1–10% of all cases of Paget’s disease with an incidence estimated at around 0.6/100 000 people per year in Europe.^21,22^ Among female patients, more than 80% of extramammary Paget’s disease are located in the vulva.^21^ Of all primary vulvar Paget’s disease cases, vulvar Paget’s disease with invasive adenocarcinoma is reported in 16–19% and vulvar Paget’s disease as a manifestation of an underlying vulvar adenocarcinoma is reported in 4–17% of all cases.^23–25^

Vulvar melanoma accounts for 6% to 10% of vulvar ;malignancies and only about 3% of all melanomas.^26–28^ An analysis of the National Cancer Database showed that melanoma in situ is less frequent than vulvar melanoma, with a median age at diagnosis of 63 and 66 years, respectively.^29^

### Molecular Etiology

VHSIL is the precursor of HPV-related invasive carcinoma and it is caused by high-risk HPVs (HPV 16 in >70% of cases),^16,30^ with smoking and immunosuppression as additional risk factors.^31^

VHSIL oncogenesis is comparable to that of high-grade squamous intraepithelial lesion (HSIL) of the cervix, vagina, and anus. Molecular heterogeneity is observed among anogenital HSIL. High host-cell DNA methylation levels in VHSIL^32^ seems to reflect a high cancer risk, which might be relevant when conservative management for VHSIL is considered. Using whole-genome shallow sequencing, a chromosome 1pq gain was identified as another strong indicator for the risk of HPV-positive VIN to progress to vulvar squamous cell carcinoma.^33^

The HPV-independent pathway is less well understood and, although approximately 80% of vulvar carcinomas in Europe are HPV-negative, less than 10% of vulvar pre-invasive lesions are differentiated VIN.^15,16^

dVIN and HPV-negative vulvar squamous cell carcinoma arise mostly in a field of lichen sclerosus or lichen planus, chronic inflammatory lymphocyte-mediated skin diseases.^34^

In dVIN TP53 mutations are frequently identified. Cyclin D1 amplification and copy number variations in chromosomes 3, 8, and 11q13 have been reported in HPV-negative VIN, similarly to HPV-negative vulvar squamous cell carcinoma.^33,35^

A subset of the HPV-independent precursors was found to be TP53 wild-type with somatic mutations in PIK3CA, NOTCH1, and HRAS suggesting a third, not-previously described, molecular subtype.^36–38^

The proteomic analysis points at inflammation as a driver of progression^39^: the chronic inflammatory environments in lichen sclerosus and lichen planus are considered the main contributory factors for oxidative damage and local immune dysregulation.^40–47^

Vulvovaginal microbiome disturbances seem also to be a trigger for the inflammatory response altering the balance in the host’s commensal microbes.^39^

Vulvar Paget’s disease type Ia is an in situ adenocarcinoma of the vulvar skin, which may give rise to invasive adenocarcinoma.^7^ Vulvar Paget’s disease arises from intraepidermal pluripotent stem cells in the infundibulo-sebaceous unit of hair follicles and adnexal structures.^7,48^ The reported frequency of HER2 oncogene amplification varies.^22,49–52^ Mutations in genes encoding the PIK3/AKT cascade have been found to significantly correlate with CDH1 hypermethylation.^53,54^ Amplification at chromosomes Xcent-q21 and 19, as well as loss at 10q24-qter, have been reported.^55^

Cutaneous and mucosal vulvar melanomas arise from melanocytes. Melanoma in situ consists of malignant melanocytes that spread along the epidermis but do not extend into the papillary dermis. Vulvar melanomas may develop de novo, or from pre-existing benign or atypical pigmented lesions. The etiology and pathogenesis are largely unknown. Ultraviolet radiations are unlikely to be involved since most tumors arise on surfaces not exposed to sun.^56^

### Clinical Aspects

There is no single pathognomonic clinical feature of vulvar SIL. Approximately 60% of patients report itching and/or irritation, pain, or bleeding along with visible vulvar lesions.^57^ In others, lesions are diagnosed incidentally during a routine gynecological examination. It is difficult to distinguish among various types of vulvar lesions based only on macroscopical aspects and the distribution of vulvar changes. Clinical aspects of vulvar SIL are variable with significant differences in number, size, shape, color, surface, thickness, and topography. Lesions may be solitary or multiple. They are characteristically papular, raised, with sharp borders and a keratotic, roughened surface. Their color may range from white to red, gray, blue, or brown. Magnification of the vulvar skin with lens or colposcope after thorough naked eye examination may allow (a) a better definition of the extent of the lesion, (b) the direction of biopsies to the area(s) of most clinically severe abnormality, and (c) direct treatment by visualizing anatomic landmarks.

Three percent to 5% acetic acid can be applied by expert hands^58^ when HPV-associated SIL is suspected: sharply demarcated and raised acetowhite epithelium generally corresponds to VHSIL, whereas dVIN generally does not react to acetic acid. It should be kept in mind that acetic acid in vulvoscopy should be used only in experienced hands, considering the high false-positive rate.^58^

**FIGURE 1:**
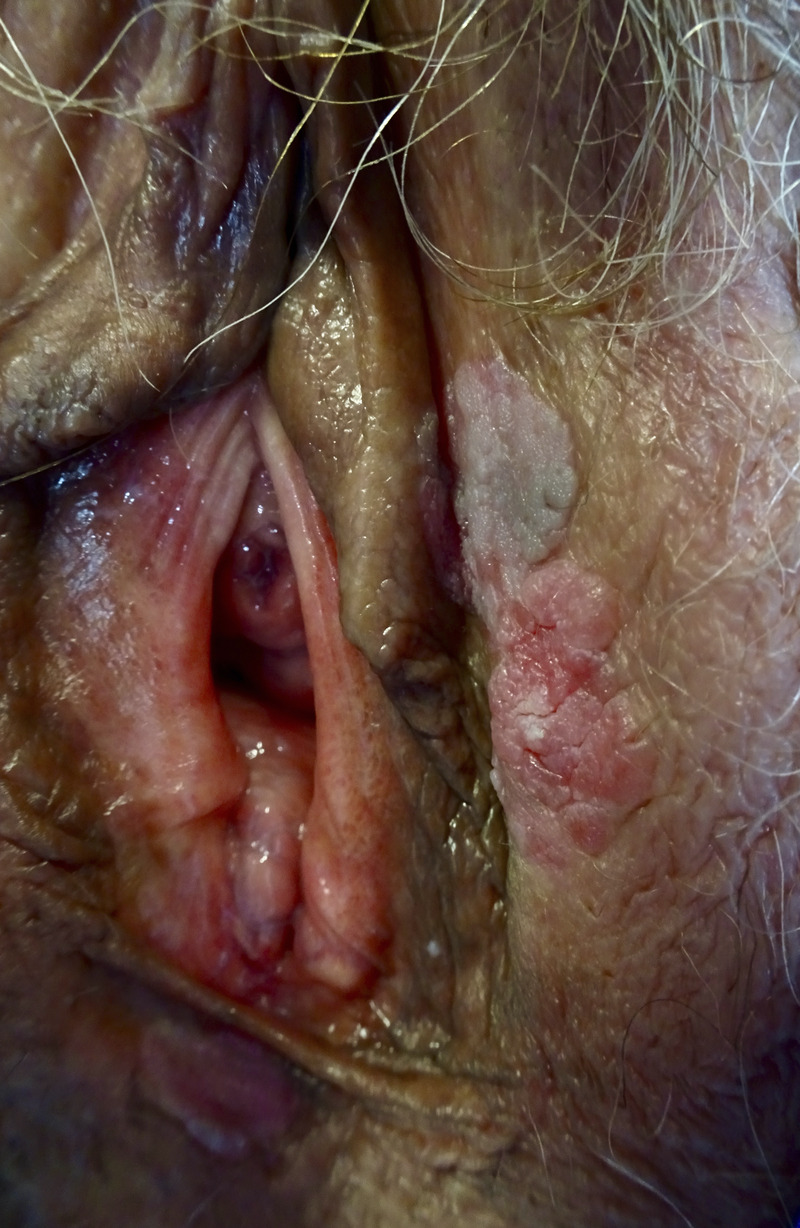
Vulvar high grade squamous intraepithelial lesion; brownish and erythematous poorly marginated plaques on the inner side of left labium.

VHSIL tends to occur in young women and it is usually multifocal, located around the introitus, and often involving the labia minora (Figure 1). Multicentric/multizonal disease often presents in cases with VHSIL, and may involve cervical, vaginal, perianal, or anal squamous epithelium. A careful examination of the whole vulva, perineum, perianal, and anal areas, including the cervix and vagina, is mandatory. There are not enough data to screen all VHSIL patients with high-resolution anoscopy, and anal cytology sensitivity seems to be low in women with VHSIL.^59^ In the meantime, accurate anal squamous cell carcinoma symptom questioning should be performed in this group of patients.

**FIGURE 2:**
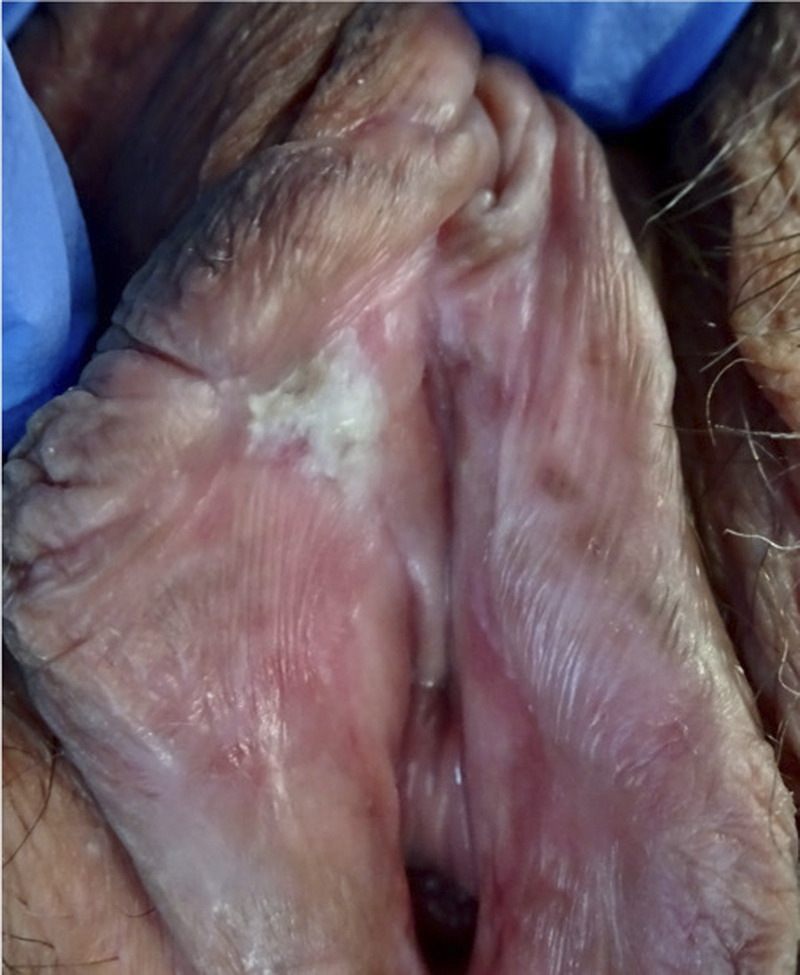
Differentiated vulvar intraepithelial neoplasia; whitish poorly marginated plaque on internal side of right labium minus in a field of lichen sclerosus.

The clinical approach to dVIN patients is completely different in that it is seen primarily in older women (median age 67.0 years vs 47.8 years in VHSIL).^15,17^ Clinically, dVIN is sometimes difficult to distinguish from the associated dermatosis, in particular lichen sclerosus involving the adjacent skin, and usually it appears as unifocal and unicentric poorly demarcated pink or gray-white (hyperkeratotic) rough plaques^60–62^ (Figure 2). Long-lasting symptoms and treatment-resistant dermatoses need to be carefully inspected to rule out dVIN and to promptly biopsy.

An underlying early invasive squamous cancer may be present in up to 20% of VHSIL patients^63,64^ and this percentage is even higher in dVIN.

For a definitive diagnosis of a vulvar lesion, a biopsy needs to be performed. As many vulvar cancers are missed and have delayed diagnosis due to biopsies not having been taken, a biopsy should be performed of any suspicious lesion identified with multiple biopsies performed for lesions of multiple colors, large lesions, and multicentric lesions.

Punch/incision biopsy establishes the diagnosis. All multiple lesions should be biopsied separately and mapped.

#### Differential Diagnosis

Due to the variation in the clinical features of vulvar SILs, these lesions can mimic different diseases: lichen simplex chronicus, lichen sclerosus, lichen planus, psoriasis, contact dermatitis, and more.

### Paget’s Disease

Vulvar Paget’s disease is considered the great mimic of vulvar pathology. Its lesions can be mistaken for chronic dermatitis or dermatosis, and so delay the histological diagnosis of the disease. In the ISSVD *Terminology and classification of vulvar dermatologic disorders* (2011), vulvar Paget’s disease is assigned to the morphological group 2, ‘Red lesions, patches and plaques’, and to subgroup B, ‘Red patches and plaques (no epithelial disruption)’.^65^

**FIGURE 3:**
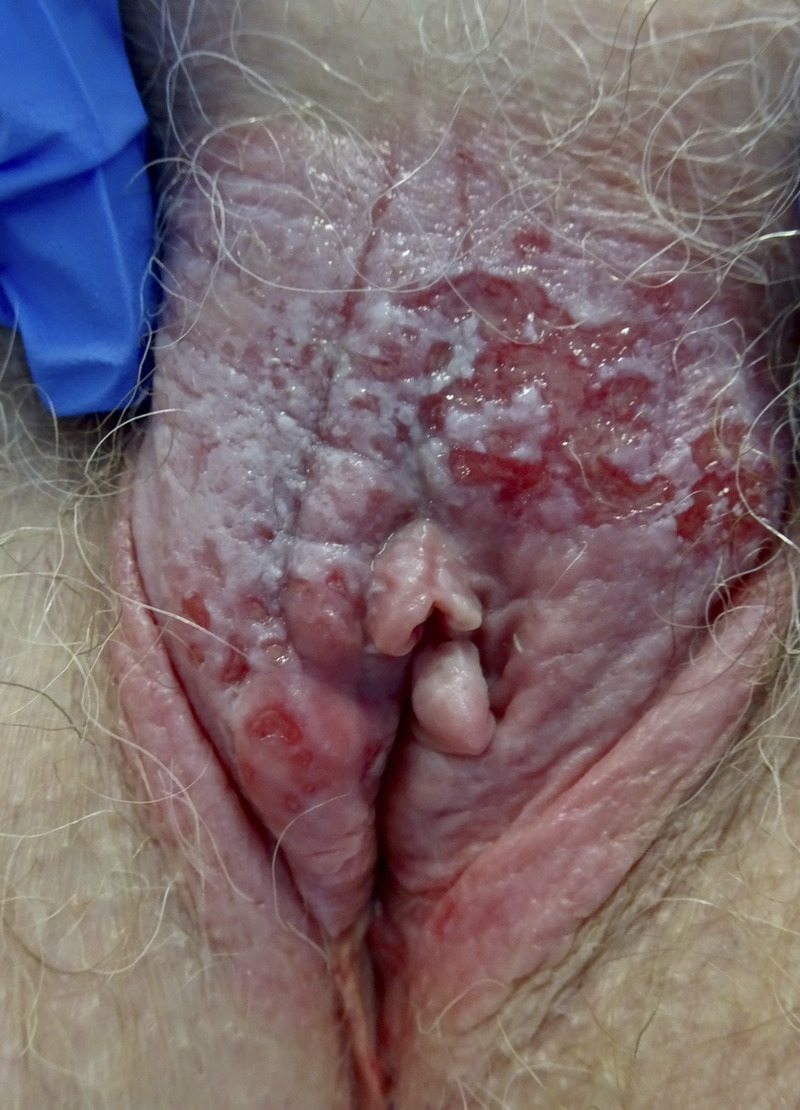
Vulvar Paget disease in situ; erythematous and white lesion involving whole vulva with superficial erosions.

On inspection, the lesion may look red or exhibit different shades of white and gray, usually eczematous, ulcerated, or with a crusty appearance, but it is seldom pigmented (Figure 3). Most of the lesions are found on the labia majora and vary in size. However, vulvar Paget’s disease can involve labia minora, clitoris, inguinal folds, urinary meatus, and perineum.^66,67^

The visible borders are mostly irregular, slightly elevated, and sharply demarcated; the disease often extends the macroscopic margins. With periurethral and perianal lesions, an involvement of the skin by a non-cutaneous underlying neoplasm must be excluded.

#### Differential Diagnosis

Lichen sclerosus, dermatophytosis, candidiasis, contact dermatitis, psoriasis, seborrheic dermatitis, and squamous VIN are among the differential diagnoses. Finding similar lesions elsewhere on the body and a biopsy including the derma with appropriate use of immunohistochemistry will confirm a vulvar Paget’s disease diagnosis.

### Melanoma in Situ

**FIGURE 4:**
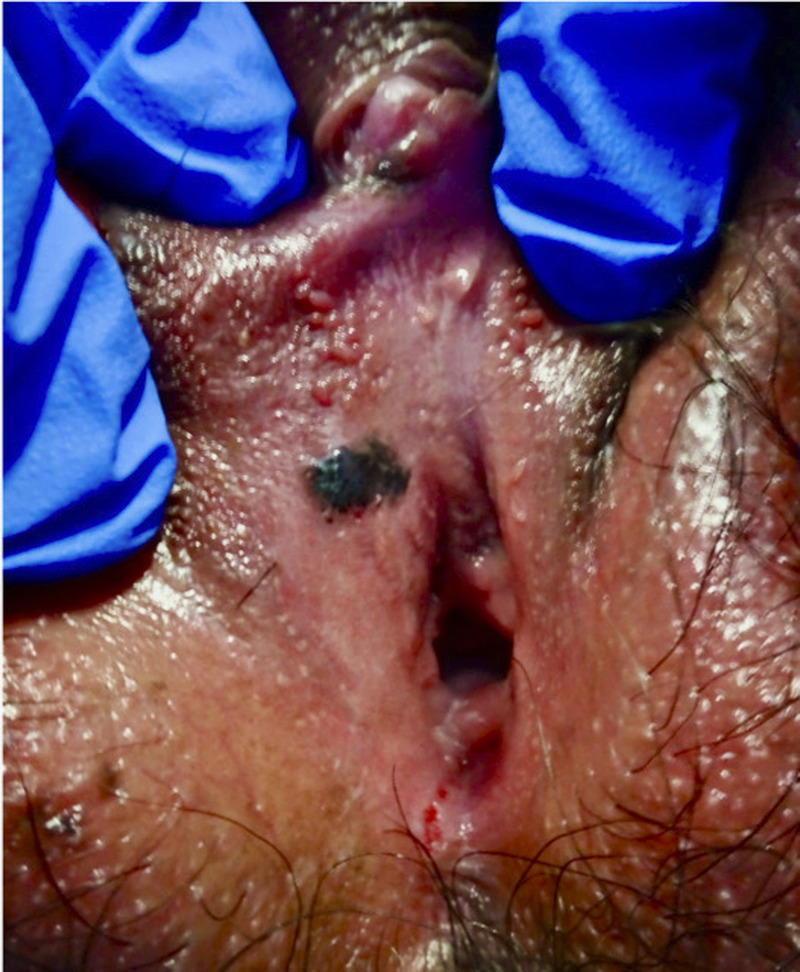
Melanoma in situ; black poorly marginated oval smooth lesion on the right superior vestibule.

Biopsy including the derma allows diagnosis of melanoma in situ, which is an uncommon pigmented vulvar lesion often clinically indistinguishable from the more common benign pigmented lesions, such as melanosis (Figure 4). Asymmetry, indistinct borders, variegated color, and a large diameter (>6 mm) are similar in both lesions. Consequently, a biopsy is necessary for diagnosis, and the threshold to biopsy a genital pigmented lesion should be low.^68,69^

#### Differential Diagnosis

Physiologic hyperpigmentation, congenital adrenal hyperplasia, Addison’s or Cushing’s disease, postinflammatory hyperpigmentation, acanthosis nigricans, seborrheic keratosis, vulvar melanosis/ lentiginosis, melanocytic nevi (pigmented nevi, nevocellular nevi, common nevi), pigmented condylomata acuminata, pigmented basal cell carcinoma, pigmented VIN, and squamous cell carcinoma should be considered among the differential diagnoses.

### Histopathology

Accurate histological diagnosis is crucial for appropriate treatment; histological assessment of vulvar intraepithelial lesions requires pathologists dealing with high-volume vulvar biopsies. Interobserver agreement was demonstrated low for VHSIL^70^ and it is even worse for dVIN diagnosis^71,72^ where associated dermatoses complicate the histological pattern.^73^

The recommendation for tissue sampling of suspected precursor lesions is to obtain optimal specimens with a minimum 4 mm width with 5 mm depth for hair-bearing skin and 3 mm depth for hairless skin and mucosal sites, achieved with punch, cold knife, or suture-assisted snip. In the case of ulcer or fissure, biopsy should be performed where there is intact epithelium.^74^

In non-invasive lesions of the vulva, immunohistochemistry is helpful in distinguishing difficult cases (Table 1).

**TABLE 1:** Immunohistochemistry in Vulvar Pre-invasive Lesions

Lesion	Immunohistochemistry	Comment
VHSIL (VIN 2/3)	P16 block positivity, ki-67 extends above basal layers through entire epithelium	Ki-67 will stain above the basal layers in LSIL as well and cannot be used to distinguish LSIL from VHSIL. P16 is more useful in this distinction and can be occasionally positive in LSIL
dVIN	Aberrant p53 staining patterns. P16 not block positive. Ki-67 confined to basal layers	A panel of p53, p16, and ki-67 helpful in distinguishing VHSIL from dVIN
Vulvar Paget’s disease	Cells contain mucine (PAS-D or alcian blue), mucicarmine, CK 7, GCDFP-15, GATA3^77^	Stains to distinguish secondary Paget’s disease of urothelial (including uroplakin^78^) or anorectal origin (including CDX-2, CK20^79^) should be considered in appropriate cases
Melanoma in situ	Positivity with s100, Melan-A, and HMB 45^80^	A panel to distinguish melanoma in situ from Paget’s disease can be helpful

dVIN, differentiated-type vulvar intraepithelial neoplasia; LSIL, low-grade squamous intraepithelial lesions; VHSIL, vulvar high-grade squamous intraepithelial lesions.

**FIGURE 5:**
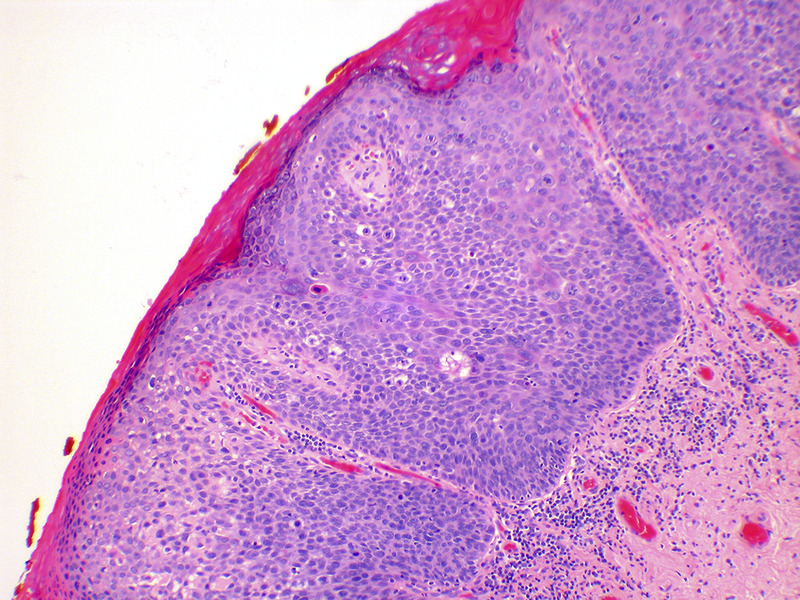
Vulvar high-grade squamous intraepithelial lesion; the lesion shows full thickness abnormality of maturation, and acanthosis (hematoxylin and eosin, x 10 magnification).

VLSIL shows abnormal maturation and dysplastic features up to the lower third of the epithelium, while in VHSIL these abnormal features extend above the lower third of the epithelium (Figure 5). Immunohistochemistry with p16 can be of help to distinguish VLSIL from VHSIL, or atrophy from VHSIL, as VHSIL shows block positivity compared with mimics.^3^

The histologic features of dVIN can be subtle, and the histological diagnosis may be further complicated by coexisting conditions such as lichen sclerosus. dVIN underdiagnoses could be partially explained by misclassification as reported by Van de Nieuwenhof et al, who found that 42% of the biopsies initially diagnosed as lichen sclerosus were reclassified as dVIN after review.^73,75^

**FIGURE 6:**
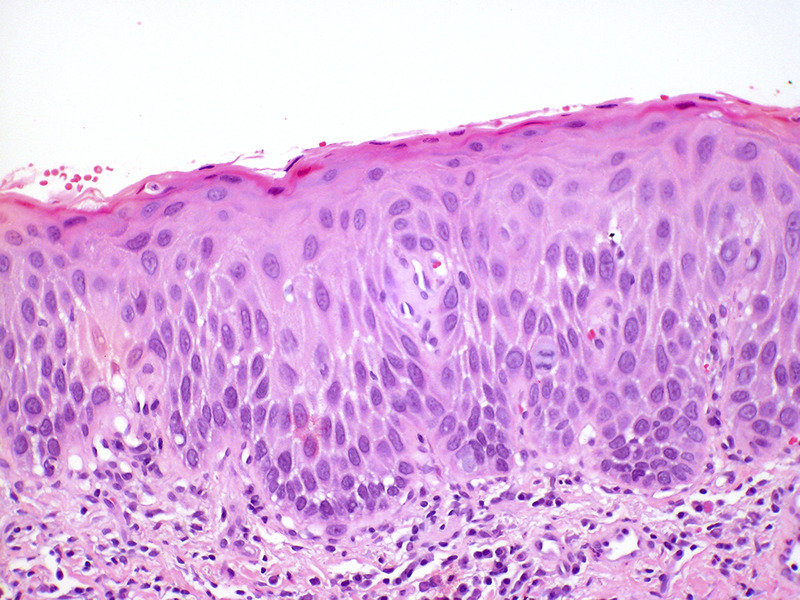
Differentiated vulvar intraepithelial neoplasia (dVIN). The histologic changes of dVIN are very subtle, and may be missed. Here there is basal atypia and acanthosis, but overall maturation is maintained. P53 and Ki-67 showed increased basal activity, and p16 was not block-positive, not shown (hematoxylin and eosin, x 20 magnification).

dVIN shows basal atypia with abrupt (premature) maturation (hypereosinophylic keratinocytes), basal spongiosis, absence of granular layer, and parakeratosis (Figure 6). Nuclear atypia with enlarged and angulated hyperchromatic nuclei and increased mitotic activity together with premature keratinization with hypereosinophylic keratinocytes may be seen. Other common features in dVIN are squamous hyperplasia with elongation of rete ridges and pronounced intercellular bridges in the lower part of the epithelium and absence of the granular layer in combination with hyperkeratosis with parakeratosis. P53 often shows an aberrant staining pattern in the dysplastic cells of dVIN.^38,74,76^

**FIGURE 7:**
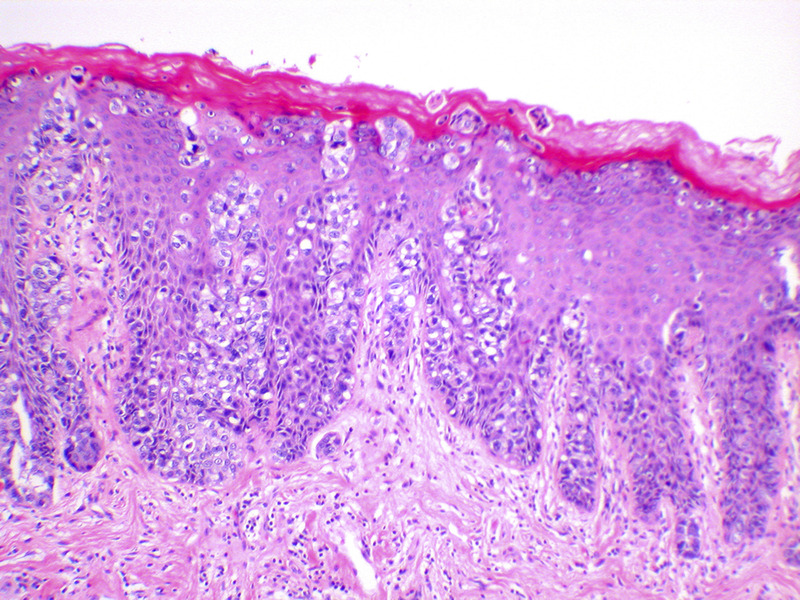
Vulvar Paget disease. The large cells of Paget’s disease are seen predominantly in the basal epithelium, but percolate up the epithelium in what is known as ‘Pagetoid spread’. Negative markers of melanoma, and positive markers of Paget, such as periodic acid Schiff stain with diastase (PAS-D), and breast markers such as Gata-3, are helpful in making this diagnosis (hematoxylin and eosin, x 10 magnification).

Vulvar Paget’s disease is usually an *intraepithelial* lesion. Histologically, the Paget cells are seen predominantly at the dermal– epidermal junction, percolating up the epithelium as individual cells in what has been called ‘Pagetoid spread’ (Figure 7). Paget cells are large and have prominent eosinophilic, basophilic, amphophilic or clear cytoplasm and vesicular nuclei with prominent nucleoli.^77^

**FIGURE 8:**
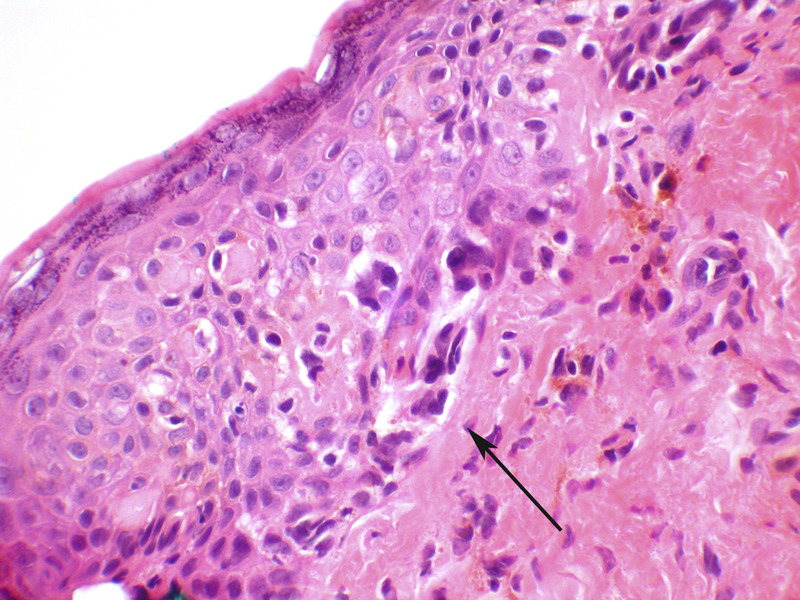
Melanoma in situ. Atypical melanocytes are seen predominantly in the basal portion of the epithelium (arrow) and will stain for melanocytic markers, which helps distinguish this lesion from Paget’s disease, which can be architecturally similar. This lesion did show pigmentation (hematoxylin and eosin, x 40 magnification).

Melanoma in situ of the vulva is rare.^67^ It must be distinguished from Paget’s disease, as the atypical melanocytes arise at the dermal–epidermal junction, as individual cells and clusters, and spread upwards in the epithelium by ‘Pagetoid spread’ (Figure 8). Melanoma in situ will stain for markers of melanoma, including s100, Melan-A, and HMB 45.

### Immunology

The promising clinical results of immunotherapy in VHSIL treatment proceeded in parallel with the studies on immunology and VHSIL microenvironment.^81,82^

Persistent HPV infection in VHSIL is able to induce a local immunosuppressive microenvironment, with upregulation of T-regulatory cells, increased infiltration with CD4+ (T helper cells), and decreased number of CD8+ (cytotoxic T cells).^81–83^

The presence and clinical impact of different myeloid cell populations in patients with non-recurrent and recurrent VHSIL were studied,^84^ showing the highest number of intraepithelial CD14+ (marker for monocytes) in the non-responding group. In VHSIL the population of M2 macrophages exceeds the M1 macrophages by at least four times, suggesting an immunosuppressive environment in the VHSIL epithelium.^84^

Some VHSIL lesions are infiltrated by high numbers of regulatory T cells (Tregs) that may induce an immunosuppressive microenvironment.^85^ Clinical response to immunotherapy in VHSIL is associated with an increase in intralesional CD8 + T cells as well as low numbers of Tregs.^81,86^ Indeed normalization of CD4+, CD8 + T cell counts in the epidermis and clearance of HPV is correlated with histological regression of VHSIL.^81^

HPV clearance after VHSIL treatment with imiquimod was also associated with a decreased number of intraepithelial CD14 + cells and an increased number of CD1a + Langerhans cells.^81^ On the other hand, the increase in CD14 + myeloid cells characterizes a progressive course of vulvar neoplasia^87^ and it is an independent prognostic factor for decreased recurrence-free survival.^84^

Complete responders to HPV therapeutic vaccination showed significantly stronger response of interferon (IFN)-γ-associated proliferative CD4 + T cells and a broad response of CD8 + IFN-γ T cells than did non-responders.^88,89^

Thus, an estimation of the number of intraepithelial immune cells may help in stratifying the prognosis of patients diagnosed with VHSIL and serve as a predictive biomarker for clinical response of VHSIL to immunotherapy and therapeutic vaccination.

Tumor microenvironment in vulvar Paget’s disease has been scantly studied. Tregs in vulvar Paget’s disease are frequently found at the epidermal–dermal junction,^90^ while healthy surrounding skin is negative for Tregs. Increased Tregs infiltrate was associated with more frequent positive surgical margins and recurrence of disease.^91^

It has been hypothesized that this is due to both local immunity suppression and lack of recognition of the Paget cells by the immune system as malignant or aberrant cells.^92^

Future research will focus on the changes in the immune infiltrate in vulvar Paget’s disease, clarifying clinical outcomes after imiquimod treatment.

### Management

#### Vulvar Squamous Intraepithelial Lesions

For dVIN, an excisional procedure must always be adopted.

For VHSIL, both excisional procedures and ablative ones can be used. The latter can be considered for anatomy and function preservation and must be preceded by several representative biopsies to exclude malignancy.

Medical treatment (imiquimod or cidofovir) can be considered for VHSIL.

In the past, extensive surgery with the intent to eradicate disease was the standard of therapy. The current aims are now prevention of progression to vulvar squamous cell carcinoma, preservation of normal anatomy, symptom relief, and maintenance of quality of life and sexual function with individualized treatments.

In a long-term follow-up study, median progression time to cancer ranged from 0.3 to 24.2 years after VIN diagnosis: 4.1 years for VHSIL and 1.4 years for dVIN.^15^ A 2016 Cochrane review reported a rate of progression to squamous cell cancer in 15% of women treated surgically for VHSIL over a median of 71.5 months.^93^

The increased risk of women with vulvar squamous cell carcinoma arising in a field of lichen sclerosus (through a dVIN pathway)^94–96^ is reduced by treatment with high-potency topical corticosteroids^94,97^ and should be recommended in these patients.

### Surgical Interventions

Because of the risk of progression to invasive vulvar squamous cell carcinoma from dVIN with a short interval,^17^ there is no role for medical treatment or ablation of dVIN, and therapy is conservative excision with negative surgical margins followed by continuous follow-up.^98,99^

Surgical interventions for VHSIL include both surgical excision (from wide local excision to superficial vulvectomy) and ablative therapy (carbon dioxide (CO_2_) laser vaporization, argon beam coagulation, cavitational ultrasonic surgical aspiration). Choosing the latter treatment must be preceded by representative biopsies to exclude malignancies before treatment as there is a risk of unexpected stromal invasion.^63^ In case of positive margins after surgical excisional treatment of VHSIL, if clinical inspection does not show a residual lesion, patients must be followed, and immediate re-excision is not recommended. Surgeries resulting in significant impairment should be discouraged and, when it is occasionally necessary to perform a large resection, the use of reconstructive techniques in experienced hands is required.

Despite treatment, VIN recurrence rate ranges from 6% to 50% post treatment,^14,100–121^ and it is influenced by margins status, duration of follow-up, patient-related factors (multifocality of disease, immunosuppression, and smoking), and VIN type (even if disease outcome between VHSIL and dVIN is not always detailed). In addition, methodological limitations and statistical analysis differences between studies contribute to the wide range reported. Fifty percent of recurrences are reported within 16.9 months requiring closer follow-up during the first 2 years after surgery, particularly in patients over the age of 50.^114^

In this context, the duration of follow-up is fundamental when comparing the reported rates of recurrence: 6.8% at the 6 month mark^104^ and up to 50% by the 14th year of follow-up.^14^ Immunosuppression exemplifies another important confounding factor both for recurrence (51.5% in HIV+ vs 27% in HIV− over 32 months) and progression to invasion (15.2% HIV+ vs 1.6% HIV− over median 32 months follow-up).^101^

No randomized controlled trials were performed comparing surgery with CO_2_ laser vaporization, and the available clinical data provided low-quality evidence. Leufflen et al reported 91.0% recurrence-free survival at 1 year for surgery and 65.2% for the laser vaporization groups (p < 0.01).^109^ The mean time to recurrence following either treatment was 21.7 months. With a median follow-up of 4.4 years (range 0.8–18.4 years), the rate of progression to invasive disease was 2%.

Hillemanns et al reported a recurrence rate of 40.4% for CO_2_ laser vaporization compared with 41.7% for cold knife excision, 48.1% for photodynamic therapy, and 0% for vulvectomy, with a mean follow-up of 53.7 months.^106^

Van Esch et al reported a lower recurrence rate of surgically treated women (48.8%) compared with patients treated with laser ablation (56.0%) or combined laser and excision (66.7%).^116^ Also, Wallbillich et al reported a higher recurrence rate associated with laser ablation (45%) compared with cold knife excision (26.7%).^119^

Fehr et al^103^ and Van Esch et al^116^ reported a rate of progression of 6.1% and 15.1%, respectively, with mean time to invasion of 82 months^103^ and 71.5 months.^116^ The type of first treatment showed no differences in progression-free survival in the univariate Cox analysis.^116^

Only one paper compared^117^ loop electrosurgical excision procedure (LEEP, n = 20), cold knife surgery (n = 22), and laser vaporization (n = 20): recurrences after the first procedure were significantly fewer with LEEP (15%) and wide local excision (10%) than with laser ablation (50%).

Argon beam coagulation was evaluated in VIN3 (VHSIL) treatment, with a recurrence rate of 48.3% and a mean time to recurrence of 23.2 months.^108^ The main advantage of this treatment modality is preservation of vulvar anatomy and the ability to perform multiple treatments.

CO_2_ laser vaporization was compared with cavitational ultrasonic aspiration (CUSA) in a single randomized controlled trial.

No statistical difference in recurrence was reported at 12 months follow-up, with CUSA being reported as causing less pain and less scarring than laser.^118^ Investigating CUSA alone in VIN treatment, a recurrence rate of 35% after a median interval of 16 months and a progression rate of 3% after 33 months of median follow-up was reported.^111^

### Medical Interventions

Medical therapy is a therapeutic option suitable for VHSIL to preserve normal vulvar anatomy and to avoid mutilation. On the other hand, medical therapies do not provide histological specimens with the risk of missing early invasion foci. Consequently, several biopsies are needed prior to medical treatment.

Imiquimod is an immune response modifier directed to TLR-7 and stimulates dendritic cell secretion of pro-inflammatory cytokines, thereby eliciting strong immune infiltration.^122^ After 87% complete or partial response in patients enrolled in a pilot study,^123^ two randomized controlled trials^124,125^ compared imiquimod with placebo. The complete response for imiquimod-treated women was 81% for Mathiesen et al^124^ and 35% for Van Seters et al^125^ from 2 to 5 months after treatment. Only Van Seters et al^125^ reported 12 months follow-up data with 35% complete responders (n = 9) in the imiquimod arm compared with 0% in the placebo group; and no difference in rates of progression to invasive disease between the two arms (1/26 vs 2/26). Long-term follow-up of the initial cohort from Van Seters was available^126^ and eight out of nine initial complete responders were disease-free after a median follow-up period of 7.2 years. The lesion sizes of long-term complete imiquimod-responders were significantly smaller than those of patients with residual and/or recurrent disease.

One randomized controlled trial with 180 patients enrolled evaluated topical 5% imiquimod cream versus 1% cidofovir gel and found no difference in terms of complete response (46% for both arms).^127^ At 12 months follow-up, the complete responders showed sustained results in 87% of cidofovir complete responders and 78% in the imiquimod arm. After 18 months follow-up of the same group of patients,^128^ cidofovir complete responders had a 6% recurrence rate compared with 28.4% of the imiquimod arm.

HPV E2 DNA methylation demonstrated to be a predictive biomarker for successful response in VIN treatment with cidofovir.^129^ Two other non-randomized controlled trials of imiquimod as single therapy were available and reported a range of recurrence 20.5–27% after 16–21 months of follow-up.^130,131^

Combining cold knife surgery and imiquimod cream as adjuvant does not seem to offer advantages in terms of lower recurrence rate,^105^ but may allow less extensive excisions and better preservation of the anatomy and function.

### Photodynamic Therapy

Photodynamic therapy uses a topical photosensitizer, 5-aminolevulinic acid, in combination with non-thermal light of appropriate wavelength to induce oxidation reactions that lead to cell apoptosis. The overall clinical response varies from 31.2% to 56%,^86,121,132^ and it seems to be comparable to laser ablation.^132,133^

The recurrence rate ranges from 14.3%^132^ at a mean 13 months to 48%^106^ after a mean 53.7 months of follow-up. Only one paper reported a 9.4% rate of invasion after treatment.^86^

### Therapeutic Vaccine

Therapeutic vaccine against HPV-16 E6 and E7 oncoprotein has been investigated, and an observational phase II study showed promising results.^88^ At 12 months of follow-up, 47% of patients showed complete response and 32% partial response; complete responders were still free of disease at 24 months.

### Follow-Up of Women With Vulvar Intraepithelial Neoplasia

Following treatment of VIN, women should be seen on a regular basis for careful clinical assessment, including biopsy of any suspicious area. Follow-up should be modulated according to the risk of recurrence (type of lesion, patient age and immunological conditions, other associated lower genital tract lesions).

The reported risk of progression to malignancy varies widely but appears to be around 10% for VHSIL and up to 50% in dVIN.^13–15,134^

The risk is higher in untreated women. Age (HR 2.3, 95% CI 1.5 to 3.4) and lichen sclerosus (3.1, 95% CI 1.8 to 5.3) are also independent risk factors for progression.^15^ Women treated surgically for VIN still have a residual risk of developing invasive cancer in the order of 2–4%.^13^

The risk for recurrence of VIN is up to 60%, independent of the surgical approach.^14^ About 25% of recurrences are late (more than 44 months after initial diagnosis) in one large long-term observational study.^114^ Women need clear information regarding signs and symptoms (such as pain or ulcers) that should prompt an earlier review. There is less evidence on long-term clinical outcomes and the risk of invasion following a full clinical response to topical medical treatments, but it may be similar to surgical treatment.

At least 4% (up to 25%) of women diagnosed with VIN will have intraepithelial neoplasia at other lower genital tract sites,^135,136^ and accurate inspection of lower genital tract sites including cervix, vagina, vulvar, and perianal skin is mandatory during follow-up. Similar rates of VHSIL were found in one study whether or not the woman had a previous hysterectomy, indicating that surveillance of the vagina is still required.^137^ Initiatives for anal squamous cell carcinoma screening in HPV-related VIN and vulvar squamous cell carcinoma patients are needed.^19^

Data suggest that dVIN carries a higher risk of progression and recurrence than VHSIL^62,73^ and closer follow-up is recommended after dVIN treatment.

## VULVAR PAGET’S DISEASE

Recent studies favor an approach of using imiquimod. Surgery must take into consideration that the extension of the disease is usually wider than what is evident in the skin. A 2 cm margin is usually considered necessary.

Surgery is the cornerstone of vulvar Paget’s disease treatment in the published literature (ranging from 58.6% to 100% in published papers). Surgical options vary from local wide excision to radical vulvectomy with or without inguinal lymphadenectomy. If there is no underlying invasive disease (intraepithelial disease; 1 a), a wide resection with 2 cm clear margins is the most reported surgical treatment. Frozen section may be useful to achieve margin-free surgical excisions as disease often extends past what is visible to the eye.^138–141^ However, there is no clear demonstration that there should be a minimal distance to resection margins for vulvar Paget’s disease and the level of evidence is not very high to support this statement. Re-excision to achieve larger margins with ‘mutilation’ could not be of benefit. In cases with invasive disease or an underlying adenocarcinoma, a more radical approach (both in extension and in depth of excision) should be considered^138,140^ with lymphadenectomy,^138,140,142^ as there are not enough data for sentinel node in invasive vulvar Paget’s disease.

Topical 5% imiquimod cream has also been shown to be a safe conservative treatment option for in situ vulvar Paget’s disease with minimal adverse effects. Complete response rates have been reported with a range from 22% to 90% of cases.^22,143,144^ This allows a chance for the anatomical and functional conservation of vulvar structures. Treatment schedule varies among different studies (1–5 times a week, from a minimum of 3 weeks to an entire year). A total treatment duration of 16 weeks seems to be commonly used.^22,143^

Photodynamic therapy is not curative at all but can be used for symptom control.^145^

Radiotherapy can be considered when there is lymph node positivity or positive surgical margin in situations with associated invasive disease where there are contraindications for surgery or inoperable situations. There has still been no standard dose or schedule for the radiotherapy, so larger case series are warranted.

## MELANOMA IN SITU

A wide local excision with 1 cm free surgical margins is recommended.

Melanoma in situ is rarely seen in the vulva and appears to progress gradually to invasive melanoma.^146,147^ In some reports, association with lichen sclerosus is detected during the in situ phase, which usually disappears at later invasive stages.^148^

An excisional biopsy is the preferred method for diagnosis in small lesions with complete excision and depth to rule out invasion.^149^ A punch biopsy can also be used for large lesions, targeting the thickest area of the lesion.^149,150^ A wide local excision with 1 cm free surgical margins is considered curative.^151^ There is no need for lymph node assessment. Prognosis is usually excellent, being slightly better for melanoma in situ developing from melanocytic nevi, compared with those de novo.^152^

Only one study reported details of patients with vulvar melanoma in situ. The study evaluated 394 patients with a median age of 63. The 5 year overall survival rate was 74.4%. Vulvar melanoma in situ and invasive melanoma show worse overall survival compared with non-vulvar melanomas.^28^

### Prevention

Most of the vulvar LSIL and VHSIL are HPV-related; the predominant HPV types are HPV 6 and 11 in LSIL, HPV 16 in VHSIL,^153^ and HPV 16 and 33 in HPV-related invasive vulvar cancer.^16^ The HPV vaccines are highly effective in preventing lesions related to the vaccine types.^154,155^ Approximately 90% of these lesions are related to HPV genotypes included in the 9-valent HPV vaccine.

Women with HPV-related vulvar disease are at high risk for contracting subsequent or recurrent disease.

Published studies show reduced VHSIL recurrence when HPV vaccines are administered before or after treatment^156,157^; HPV vaccination may be beneficial, and further studies are necessary to support these findings. Early prophylactic vaccination is recommended to every girl and woman according to national guidelines. Women with lichen sclerosus showed a risk of cancer of 3.5% (incidence rate of 8.1:1000 person-years), increasing with advancing age.^158,159^ A recent Dutch study analyzing the incidence rate of vulvar squamous cell carcinoma in patients with VIN (median follow-up time 13.9 years, range 0.3–27.4 years) demonstrated in multivariate Cox regression analysis that type of VIN, age, and lichen sclerosus were independent risk factors for vulvar squamous cell carcinoma, with hazard ratios of, respectively, 3.0 for dVIN (vs VHSIL), 2.3 for age > 50 years (vs <50 years), and 3.1 for lichen sclerosus (vs no lichen sclerosus).^15^

Women with lichen sclerosus who are compliant with topical steroid use have a much lower rate of vulvar cancer and better symptom control.^97^ The current belief is that women should continue regular use of topical steroids, even if asymptomatic, at least weekly and have lifelong regular check-ups (at least every 6–12 months, or when symptoms do not improve with adequate treatment, or new lesions are identified). Well-controlled patients can have these follow-up visits with their primary care physicians.^160^ Long-term follow-up is also advised for those who had the diagnosis during childhood, even if they experienced significant improvement during adolescence.^161^ No response to treatment or suspicious lesions (persistent erosions, tumors, and hyperkeratosis) should promptly be biopsied. Women with vulvar cancer and lichen sclerosus are often not offered topical steroids post-treatment of the cancer, but their use may reduce the recurrence risk to nearly a half (27% vs 44–47%).^94^

### Immunosuppressed Patients

The immunosuppressed population includes HIV-infected women, solid organ transplant recipients, as well as women undergoing immunosuppressing treatments for rheumatologic or autoimmune diseases. Evidence suggests that immunosuppression is a risk factor for development of HPV-related pre-invasive lesions and invasive cancers.

HPV and HIV have tight immune interactions, the latter facilitating HPV infection through the disruption of epithelial tight junctions.^162^

In addition, immune system defects such as CD4 + lymphocyte loss may contribute to impaired clearance or reactivation of latent HPV infections.^162,163^

HIV-infected women have higher incidence rates of VIN at a younger age and frequently have multifocal and multicentric HPVrelated lesions.^101,110,164–167^ Indeed high-grade cervico-vaginal cytology was reported following treatment for VIN or vulvar cancer with OR 3.4 for immunodeficiency (95% CI 1.3 to 8.8).^135^

The recurrence and progression rates are far higher and with a shorter disease-free interval for HIV+ women than HIV− women,^101,165^ with a lower CD4 + lymphocyte count linked to shorter time to recurrence.^110,165^ Highly active antiretroviral therapy may decrease the incidence of condyloma and LSIL but appears to have no impact on VHSIL.^168–170^

Immunosuppressive drugs for renal transplant recipients may increase the risk of HPV carcinogenesis.^171,172^ Renal transplant recipients are at higher risk of VHSIL within 20 years after transplantation (5–12% vs 0.2–0.4% of female non-renal transplant recipients).^173^ One systematic review reported a higher SIR of HPV-associated cancers in transplant patients compared with the general population: 2.1 (95% CI 1.37 to 3.30) for cervical cancer, and 22.8 (95% CI 15.8 to 32.7) for vulvar and vaginal cancer.^174^ A 41-fold increased risk for vulvar cancer and a 122-fold increased risk for anal cancer among renal transplant recipients were also reported in a Dutch study. Interestingly, 100% of vulvar cancer in this population were HPV+, compared with as low as 4.9% in immunocompetent patients.^175–178^

Thus, immunosuppressed patients should undergo a complete lower ano-genital tract examination as a part of routine screening and be appropriately managed by the multidisciplinary team.

### Education and Information

The adherence to follow-up after VHSIL treatment is essential, due to the risk of recurrence; however, no study was performed with this aim. Thus, there is no evidence about effective interventions for enhancing patients’ adherence to follow-up. Providing patients oral and written information on their medical situation appears, however, to be justified as it might improve patients’ awareness of symptoms and the need for regular clinical vulvar examination.^179^

When considering patients’ adherence to prescribed medication, current intervention methods seem to be not very effective, but are likely to be more successful when repeated.^180,181^ This suggests that information delivered to these affected patients should be multimedial, using various supports, and repeated over time.

### Reconstructive Surgery

Limited evidence is available regarding indications for reconstructive surgery and procedure selection for patients diagnosed with vulvar precancer lesions, and generally comes from retrospective, observational, and descriptive studies.^93,182^

Therefore, patients should be consulted before surgery by a team experienced in the field of vulvar and reconstructive surgery, with all members using consistent terminology based on well-defined and reproducible anatomic landmarks.^183^ In general, premalignant vulvar lesions are excised in a conservative fashion, preserving as much of the vulvar anatomy and function as possible. Surgery ranges from a local excision to skinning (superficial) vulvectomy with the removal of the clitoral hood. The majority of wounds after being locally excised, if not distorting the local anatomy, are closed primarily and do not require reconstructive surgery. The larger the size of the excision of a vulvar premalignant lesion, the more the quality of life and sexual function decreases without reconstruction.^184^ Therefore, the method of reconstruction should be individually tailored to the size and site of the vulvar defect. Reconstructive procedures are aimed at tension-free skin closure, maintenance of vulvovaginal anatomy, and appearance without shrinkage of vaginal and urethral introitus. It is important to avoid their lateral displacement and preserve cosmesis, sensation, and sexual function.^185^ Skills in basic plastic surgery procedures are consequently required.

Where a primary closure without tension is not possible, the defect may be closed by rotated or transposed local cutaneous flaps, although wound size exceeding 5 cm might be a limiting factor.^186–188^

Superficial (skinning) vulvectomy with subsequent grafting of split or full thickness skin can be applied in a limited group of patients with confluent multifocal lesions or involving clitoris, urethra, vaginal introitus, or anus not responding to medical therapy. Skin grafts are usually taken from the groin, mons pubis, or inner thigh. Recently, dermal substitutes less prone to wound contraction and more pliable than grafts are starting to be applied in reconstructive surgery.^189^ Dermal substitutes are collagen-based regenerative matrices, either acellular or synthetic, placed in direct contact with the wound and promoting autologous and spontaneous skin regeneration. These procedures allow the preservation of the shape and functional integrity of the vulva.^190–194^

Where extensive excision is performed, traditional fasciocutaneous and myocutaneous local or regional advancement flaps remain the best choice, and more advanced perforator flaps are usually not needed.^182,188,195–198^

### Teleconsulting

Telemedicine is broadly defined as the ‘use of electronic information and communication technologies to provide and support healthcare when distance separates the patient and the healthcare professional’.^199^ In the last 30 years, this field has undergone a huge expansion and many subspecialties are trusting this type of healthcare (eg, telecolposcopy).^200^ Vulvar pathology could follow the example of tele-dermatoscopy, in which patients send digital photographs to their physician, who can examine skin lesions without visiting the patient. The follow-up of vulvar dermatoses (eg, lichen sclerosus) could be carried out using teleconsulting; some dermatologists are already doing so.^201^ Furthermore, to achieve an effective vulvar examination, patients would need to collect images of their external genitalia, improving the vulvar self-examination, which could lead to an early diagnosis and treatment of vulvar pathologies.^179^

### Quality of Life and Psychological Sequelae of Vulvar Pre-invasive Lesion Treatment

Pre-invasive vulvar lesions deserve specific attention because they affect not only functionality and body image but also psychosexual factors. Symptoms of intraepithelial neoplasia (ie, burning and itching), together with a change in appearance of vulvar skin, may cause dyspareunia and feelings of being less attractive. Additionally, concern of infecting the partner in HPV-related VIN and the potential effect on future pregnancy might contribute to the emotional burden. Surgery may exacerbate, rather than relieve, sexual dysfunction due to postoperative scarring and anxiety of revealing their body. Usually, these women have a fear of recurrence or development of cancer. Overall, a lower quality of life was reported in women with VIN.^202^ Education and psychological support by gynecologists, psychiatrists, or psychologists, together with partner counseling, could help regain sexual confidence, restore sexual functioning, and increase quality of life.

**CONSENSUS STATEMENTS**

1. In the following pre-invasive lesions of vulva, immunohistochemistry is recommended in distinguishing difficult cases: p16, ki-67 p53 (squamous lesions), PAS-D, mucicarmine, CK 7, GCDFP-15, GATA3 (Paget’s disease of the vulva), s100, Melan-A, HMB 45 (melanoma in situ).
  Consensus: 100%
2. dVIN complete surgical excision of visible lesions is recommended to treat the lesion and to exclude invasive disease.
  Consensus: 93.3%
3. After dVIN excision, treatment of associated Lichen sclerosus and Lichen Planus with topical high potency corticosteroids is recommended to reduce the risk of recurrence/progression.
  Consensus: 100%
4. Colposcopy of cervix and vagina and inspection of the entire lower genital tract, including vulvar, perianal and anal region, is recommended in women diagnosed for VHSIL.
  Consensus: 93.3%
5. Multiple representative biopsies are recommended to exclude invasion before VHSIL non-excisional treatments (medical treatment, LASER vaporization, CUSA, PDT).
  Consensus: 100%
6. Imiquimod should be considered as a therapeutic option to preserve normal vulvar anatomy in VHSIL patients.
  Consensus: 100%
7. In case of positive margins after surgical excisional treatment of VHSIL, if clinical inspection doesn’t show a residual lesion, patients must be followed, and immediate re-excision is not recommended.
  Consensus: 100%
8. HPV vaccination adjuvant to surgical treatment may be considered with the aim to reduce VHSIL recurrences.
  Consensus: 84.6%
9. In patients treated for VHSIL, life-long surveillance for HPV related carcinomas is recommended.
  Consensus: 93.3%
10. In case of positive margins after surgical excisional treatment of vulvar Paget disease, if clinical inspection doesn’t show a residual lesion, patients must be followed, and immediate re-excision is not recommended.
  Consensus: 92.9%
11. In vulvar pre-invasive lesions treatment, surgeries resulting in significant distortion of the vulvar anatomy should be discouraged.
  Consensus: 92.9%
12. After vulvar pre-invasive lesions treatment, follow up should be modulated according to the risk of recurrence (Type of lesion, patients’ age and immunological conditions, other associated lower genital tract lesions).
  Consensus: 93.3%

## Supplementary Material

SUPPLEMENTARY MATERIAL
