# Supplementary material for: The European Society of Gynaecological Oncology (ESGO), the International Society for the Study of Vulvovaginal Disease (ISSVD), the European College for the Study of Vulval Disease (ECSVD) and the European Federation for Colposcopy (EFC) Consensus Statements on Pre-invasive Vulvar Lesions
Source: J Low Genit Tract Dis. 2022 Jun 21;26(3):229–44. doi: 10.1097/LGT.0000000000000683 (PMC9232287; doi:10.1097/LGT.0000000000000683)
Supplement: SUPPLEMENTARY MATERIAL [file jlgtd-26-229-s001.docx]

A systematic literature review of the studies published between January 2000 to March 2021 was

carried out using the MEDLINE database. This search used indexing terms as follows: ablation,

administration, adult, adverse effect, adverse event, aminolevulinic acid, aminoquinolines,

anticarcinogenic agents, antineoplastic agents, antiviral drugs, antivirals, biopsy, brachytherapy,

carcinoma in situ, carcinoma in situ of the vulva, cavitational ultrasonic surgical aspiration, cavitational

ultrasonic surgical aspirator, cavitational ultrasound therapy, cidofovir, CO2 laser vaporisation, CO2 laser

vaporization, cold knife, cold knife ablation, cold knife biopsy, complications, conservative surgery,

conservative treatment, control, cryotherapy, CUSA, cytosine, differentiated vulval intraepithelial

neoplasia, differentiated vulvar intraepithelial neoplasia, excision, female, fertility, fertility outcome,

fertility preservation, fertility sparing, fertility sparing management, fertility sparing surgery, fertilitypreserving

treatment, high-grade squamous intraepithelial lesion, human papillomavirus therapeutic

vaccines, humans, gynaecological surgery, gynaecological surgical procedure, gynecologic surgery,

gynecologic surgical procedure, imiquimod, immune modulating drugs, immune modulators, indoles,

laser, laser ablation, laser therapy, loop electrosurgical excision procedure, loop electrosurgical excision

procedure conization, loop electrosurgical excision procedure specimen, medical interventions,

observation, organophosphonates, photochemotherapy, photodynamic therapy, photosensitizing agents,

postoperative complications, postoperative recurrence, pre-invasive vulval disease, pre-invasive vulvar

disease, quality of health care, quality of life, radiation therapy, radiofrequence ablation, radiofrequency,

radiotherapy, recurrence, recurrent disease, relapse, reoperation, residual disease, residual tumour, side

effects, suction, surgery, surgical intervention, surgical management, surgical outcome, surgical outcome

criteria, surgical procedures, surgical resection, surgical treatment, survival, survival rate, survival

analysis, therapeutic agents, therapeutic use, topical, topical imiquimod, toxicity, treatment, treatment

outcome, ultrasonic therapy, vaccines, vulval atypia, vulval Bowen, vulval Bowen disease, vulval

bowenoid, vulval Bowen papulosis, vulval carcinoma in situ, vulval intraepithelial neoplasia, vulval Paget

disease, vulval melanoma, vulval melanoma in situ, vulval melanosis, vulvar atypia, vulvar Bowen, vulvar

Bowen disease, vulvar bowenoid, vulvar Bowen papulosis, vulvar carcinoma in situ, vulvar intraepithelial

neoplasia, vulvar Paget disease, vulvar melanoma, vulvar melanoma in situ, vulvar melanosis.

The literature search was limited to publications in English, Italian, Spanish, Portugese, German, and

French. Priority was given to high-quality systematic reviews, meta-analyses, and randomized controlled

trials but lower levels of evidence were also took into consideration. Narrative reviews/guidelines and

ongoing trials/protocols have also been collected (MEDLINE database, Cochrane Central Register of

Controlled Trials, ISRCTN registry, ClinicalTrials (NIH), World Health Organization International Clinical

Trials Registry Platform). The search strategy excluded editorials, case reports, letters, and in vitro

studies.

A total number of 192 articles were retrieved, 89 on squamous vulvar intraepithelial neoplasia (VIN), 33 on vulvar Paget’s disease (VPD) and 26 on vulvar melanoma in situ (MIS). Further 12 articles with more than one preinvasive disease and 32 reviews were considered.

For the management section, papers with less than 20 patients were excluded.

Data extraction was performed for all articles dealing with treatment by two independent teams with double-checking.
